# Evaluation of Galactomannan Testing, the *Aspergillus*-Specific Lateral-Flow Device Test and Levels of Cytokines in Bronchoalveolar Lavage Fluid for Diagnosis of Chronic Pulmonary Aspergillosis

**DOI:** 10.3389/fmicb.2018.02223

**Published:** 2018-10-02

**Authors:** Helmut J. F. Salzer, Juergen Prattes, Holger Flick, Maja Reimann, Jan Heyckendorf, Barbara Kalsdorf, Sabrina Obersteiner, Karoline I. Gaede, Christian Herzmann, Gemma L. Johnson, Christoph Lange, Martin Hoenigl

**Affiliations:** ^1^Division of Clinical Infectious Diseases, Research Center Borstel, Borstel, Germany; ^2^German Center for Infection Research (DZIF), Partner Site Hamburg-Lübeck-Borstel, Borstel, Germany; ^3^Section of Infectious Diseases and Tropical Medicine, Medical University of Graz, Graz, Austria; ^4^Division of Pulmonology, Department of Internal Medicine, Medical University of Graz, Austria; ^5^CBmed – Center for Biomarker Research in Medicine, Graz, Austria; ^6^BioMaterialBank North, Research Center Borstel, Leibniz Center for Medicine and Biosciences, Borstel, Germany; ^7^Airway Research Center North, Member of the German Center for Lung Research, Borstel, Germany; ^8^Center for Clinical Studies, Research Center Borstel, Borstel, Germany; ^9^German Center for Infection Research, Clinical Trials Unit, Borstel, Germany; ^10^OLM Diagnostics, Newcastle upon Tyne, United Kingdom; ^11^International Health/Infectious Diseases, University of Lübeck, Lübeck, Germany; ^12^Department of Medicine, Karolinska Institutet, Stockholm, Sweden; ^13^Division of Infectious Diseases, Department of Medicine, University of California, San Diego, San Diego, CA, United States

**Keywords:** chronic pulmonary aspergillosis, bronchoalveolar lavage, galactomannan, lateral-flow device, cytokines

## Abstract

**Background:** Diagnosis of chronic pulmonary aspergillosis (CPA) is challenging. Symptoms are unspecific or missing, radiological findings are variable and proof of mycological evidence is limited by the accuracy of diagnostic tests. The goal of this study was to investigate diagnostic performance of galactomannan (GM), the newly formatted *Aspergillus*-specific lateral-flow-device test (LFD), and a number of cytokines in bronchoalveolar lavage fluid (BALF) samples obtained from patients with CPA, patients with respiratory disorders without CPA and healthy individuals.

**Methods:** Patients with CPA (*n* = 27) and controls (*n* = 27 with underlying respiratory diseases but without CPA, and *n* = 27 healthy volunteers) were recruited at the Medical University of Graz, Austria and the Research Center Borstel, Germany between 2010 and 2018. GM, LFD and cytokine testing was performed retrospectively at the Research Center Borstel.

**Results:** Sensitivity and specificity of GM testing from BALF with a cut off level of ≥0.5 optical density index (ODI) was 41 and 100% and 30 and 100% with a cut off level of ≥1.0 ODI. ROC curve analysis showed an AUC 0.718 (95% CI 0.581–0.855) for GM for differentiating CPA patients to patients with other respiratory diseases without CPA. The LFD resulted positive in only three patients with CPA (7%) and was highly specific. CPA patients did not differ significantly in the BALF cytokine profile compared to patients with respiratory disorders without CPA, but showed significant higher values for IFN-γ, IL-1b, IL-6, IL-8, and TNF-α compared to healthy individuals.

**Conclusion:** Both GM and LFD showed insufficient performance for diagnosing CPA, with sensitivities of BALF GM below 50%, and sensitivity of the LFD below 10%. The high specificities may, however, result in a high positive predictive value and thereby help to identify semi-invasive or invasive disease.

## Introduction

Diagnosis of chronic pulmonary aspergillosis (CPA) is challenging. Symptoms are unspecific, radiological findings are variable and proof of mycological evidence is limited by the paucity of diagnostic tests. Current guidelines recommend establishing diagnosis based on several characteristics including a radiological pattern consistent with CPA, proof of mycological evidence and exclusion of alternative diagnosis (Denning et al., 2016; Patterson et al., 2016). Consequently, patients usually undergo bronchoscopy to achieve both exclusion of alternative diagnosis (e.g., lung cancer or mycobacterial infection) and to obtain bronchoalveolar lavage fluid (BALF) at the primary side of infection to obtain mycological evidence.

Galactomannan (GM) testing from BALF is well established in the diagnosis of pulmonary invasive aspergillosis (IPA) (Ullmann et al., 2018), but evidence about the performance in CPA patients is limited. Only four studies have reported GM test results from BALF in CPA patients so far. Sensitivity and specificity were between 78 and 92 and 76 and 90% with an optical density index (ODI) ≥0.5, respectively (Park et al., 2011; Kono et al., 2013; Urabe et al., 2017) and 77–77% with an ODI ≥0.4 (Izumikawa et al., 2012).

The *Aspergillus*-specific lateral-flow device (LFD) is an immuno-chromatographic assay that detects extracellular glycoprotein antigen circulating in BALF secreted during active growth of the fungus (Thornton, 2008). A first clinical evaluation of the newly formatted and CE marked point-of-care LFD in BALF showed a sensitivity of 71% and a specificity of 100% for patients at risk for IPA (Hoenigl et al., 2018). Data about the performance of the LFD in BALF from patients with CPA are lacking.

Genetic association studies of genes involved in the immune response to *Aspergillus fumigatus* indicated that patients with CPA might differ from other patients in the cytokine profile, however, studies comprehensively investigating cytokines in BALF from CPA patients vs. controls are missing (Sambatakou et al., 2006; Smith et al., 2014).

We aimed to investigate diagnostic performance of GM and LFD testing, as well as various cytokine levels in BALF samples obtained from patients with CPA according to ESCMID/ERS/ECMM definition, patients with respiratory disorders without CPA and healthy individuals.

## Materials and Methods

### Study Population

Patients with CPA were recruited at the Medical University of Graz, Austria (*n* = 4) and the Research Center Borstel, Germany (*n* = 23). Healthy volunteers (*n* = 27) were recruited at the Research Center Borstel, Germany and were asked to undergo bronchoscopy for study purposes, which served as control group (Approval number: AZ 15–194). Patients with respiratory disorders without CPA infection who received bronchoscopy and BALF as part of routine clinical and subsequent microbiologic work-up due to suspicion of pulmonary infection were recruited at the Medical University of Graz, Austria (*n* = 27). Inclusion of residual BALF samples of patients was approved by the local ethics committee at the University of Lübeck, Germany (Approval numbers: AZ 12–220, AZ 14–225, and AZ 18–105) and the Medical University of Graz, Austria (Approval number: 25–221 ex 12/13).

### Case Definition

Criteria for CPA were based on the diagnostic criteria expressed by the ESCMID/ERS/ECMM guideline including a) one or more cavities with or without a fungal ball present or nodules on computed tomography scan, b) direct evidence of *Aspergillus* infection or an immunological response to *Aspergillus* spp. (e.g., *Aspergillus*-specific IgG antibody), and exclusion of alternative diagnosis, all present for at least 3 month or 1–3 month in case of subacute invasive aspergillosis (SAIA), respectively (Denning et al., 2016; Salzer et al., 2017).

### Clinical Samples

Bronchoscopies with BALF according to professional recommendations were performed in all patients at the Medical Clinic of the Research Center Borstel, Germany and the Medical University of Graz, Austria (Haussinger et al., 2004). All BALF samples were stored at -70°C and samples from the Medical University of Graz, Austria shipped on dry ice to the Medical Clinic of the Research Center Borstel, Germany, where GM, LFD and cytokine testing was performed in June 2018. BALF samples from the Medical University of Graz were, in part, published before (Prattes et al., 2014).

### *Aspergillus* Galactomannan Antigen Assay

BALF *Aspergillus* GM was determined by the Platelia EIA (Bio-Rad Laboratories, Munich, Germany) in clinical routine at the Medical Clinic of the Research Center Borstel, Germany according to the manufacturer’s instructions. BALF samples were processed in accordance to the manufactures protocol. While our analysis primarily focused on the recommended 1.0 ODI cut-off for BALF, we also evaluated a 0.5 GM ODI cut-off, following previous evidence that the 0.5 ODI cut off is preferable in patients on mold-active antifungals (Eigl et al., 2015, 2017).

### Newly Formatted Lateral-Flow Device

The newly formatted CE marked *Aspergillus* LFD (OLM Diagnostics, Newcastle upon Tyne, United Kingdom) was performed in accordance to the manufactures protocol (Hoenigl et al., 2018). Stored BALF samples were thawed, vortexed, and centrifuged for 1 min at 14,000 ×*g*. Seventy microliter of untreated BALF sample were applied to the port of the cassette, with results read 15 min later. Two interpreters read LFD test results independently without knowing the CPA status, ensuring an unbiased interpretation of the test results. Appearance of the test and the control line were considered as positive test result.

### Quantification of Cytokines

Levels of IL-1β, IL-6, IL-8, IL-10, IL-15, interferon (IFN)-γ, and tumor necrosis factor (TNF)-α were analyzed by using the Meso Scale Discovery (MSD) U-Plex Platform (MSD, Rockville, MD, Unites States). The panel was selected based on previous findings of BALF cytokines in CPA and invasive aspergillosis (Sambatakou et al., 2006; Smith et al., 2014; Heldt et al., 2017; Heldt et al., 2018). According to the manufacturer’s instructions^1^ the U-Plex linker (= binding to the U-Plex plate) was coupled to the specific biotinylated capture antibodies (= conjugated with electrochemiluminescent label), followed by the preparation of the multiplex coating solution. Fifty microliter of the multiplex coating solution were added to each 96-well U-Plex plate for a 1 h incubation. Twenty-five microliter BALF and 25 μL standard were incubated in the 96-well plate. Fifty microliter detection antibody was added for 1 h incubation to complete the sandwich immunoassay followed by adding 150 μL of 2× Reading Buffer. Each incubation was performed for 1 h on a shaker at room temperature, afterward the solutions was washed off. Analyses were performed on the MSD instrument by measuring the intensity of emitted light providing a quantitative measure.

### Statistical Analyses

Statistical analysis was performed with R Version 3.5.0. Kruskal–Wallis rank sum and pairwise Wilcoxon rank sum test were used to compare the patient characteristics and biomarker values in the three groups (patients with CPA, patients with respiratory disorders without CPA and healthy individuals). The Tukey Test was used to display the mean value differences. In this context, a power analysis was also conducted. Receiver operating characteristic (ROC) curve analyses were performed and Areas under the Curve (AUC) including 95% confidence interval were displayed to assess the diagnostic discriminatory ability of biomarkers to distinguish between CPA patients and healthy individuals and between CPA patients and patients with respiratory disorders without CPA.

## Results

A total of 81 patients were included in this analysis. Twenty-seven patients were classified as CPA and were compared to two control groups consisting of 27 patients with respiratory disorders without evidence of CPA and 27 healthy individuals. CPA patients were classified as chronic cavitary pulmonary aspergillosis (CCPA) (*n* = 12), single/simple aspergilloma (*n* = 6), *Aspergillus* nodules (*n* = 4), and subacute invasive aspergillosis [SAIA (*n* = 5)]. In more than one third of CPA patients (37%; 10/27) the diagnosis was histologically proven with exclusion of SAIA or IPA.

For all patients enough BALF sample volume was available for measurements of GM, LFD and cytokines. Patients’ characteristics and mean values for GM and cytokines as well as positive test results for the LFD are displayed in **Table 1**.

**Table 1:** Demographic data, underlying respiratory disorders and mean values with standard deviations of GM, LFD, and cytokine levels.

	All patients	CPA	Respiratory disorders without CPA	Healthy individuals	*P*-value
No. of patients	81	27	27	27	
**Sex**
- Male	48 (60.0%)	17 (63.0%)	15 (57.3%)	16 (59.3%)	0.922
- Female	32(40.0%)	10 (37.0%)	11 (42.7%)	11 (40.7%)	
Age (median. range)	59 (20–88)	63 (28–88)	66 (48–86)	29 (20–74)	<0.001^∗^
**Underlying Respiratory Disorder**
- Asthma	2 (2.5%)	2	0	0	NA
- Bronchiectasis	4 (5%)	3	1	0	NA
- COPD	32 (40%)	12	20	0	0.442
- Lung fibrosis	2(2.5%)	0	2	0	NA
- NTM	2(2.5%)	2	0	0	NA
- Prior pulmonary TB	7 (8.75%)	6	1	0	NA
- Recurrent aspirations	1(1.25%)	1	0	0	NA
- Sarkoidosis	6 (7.5%)	2	4	0	NA
- Lung cancer	6(7.5%)	2	4	0	NA
- Pulmonary embolism	1(1.25%)	1	0	0	NA
- LTOT	1(1.25%)	0	1	0	NA
- OSAS	2(2.5%)	0	2	0	NA
- Pneumonia	2(2.5%)	0	2	0	NA
**Tests**
GM (ODI mean, range)	0.69 ± 1.98	1.66 ± 3.22	0.24 ± 0.17	0.15 ± 0.05	0.006^∗^
Positive LFD	3	2	1	0	0.358
**Cytokines (pg/ml, mean, range)^#^**
- IFN-γ	102.11 ± 569.34	33.46 ± 75.10	223.77 ± 893.64	1.07 ± 0.88	0.372
- IL-10	2.410 ± 8.65	0.46 ± 0.83	5.43 ± 12.10	0.00 ± 0	0.066
- IL-15	2.29 ± 4.07	2.33 ± 3.37	3.64 ± 5.70	0.92 ± 0.70	0.059
- IL-1b	121.79 ± 507.97	113.74 ± 342.34	255.84 ± 811.74	0.74 ± 0.66	0.189
- IL-6	47.29 ± 102.45	51.38 ± 91.45	92.19 ± 140.45	0.41 ± 0.69	0.004^∗^
- IL-8	1390.34 ± 2087.14	1643.67 ± 2120.37	2545.85 ± 2369.79	24.29 ± 18.76	<0.001^∗^
-TNF-α	4.97 ± 19.64	2.00 ± 4.38	10.08 ± 29.61	0.00 ± 0	0.213


*COPD, Chronic obstructive pulmonary disease; CPA, Chronic pulmonary aspergillosis; GM, Galactomannan; IFN, Interferon; IL, Interleukins; LFD, Lateral-flow device; LTOT = Long-term oxygen therapy; NTM, Non-tuberculous mycobacteria; ODI, Optical density index; OSAS, Obstructive sleep apnea syndrome; TB, Tuberculosis; TNF, Tumor necrosis factor. ^#^For one patient in the group of respiratory disorders without CPA cytokine levels were not determined due to insufficient BALF volume. ^∗^*P*-value <0.05 statistically significant.*

### *Aspergillus* Galactomannan Antigen Assay

Depending on the cut-off used, GM antigen assay from BALF was positive in 8/27 CPA patients (30%; cut off 1.0 ODI) and in 11/27 CPA patients (41%; cut off 0.5 ODI), respectively. Neither patients with respiratory disorders without CPA nor healthy individuals had a positive GM test result from BALF with an ODI ≥ 1.0 (specificities 100% for both control groups). Two patients with respiratory disorders without CPA had a positive GM test result from BALF with an ODI ≥ 0.5, but none from the healthy individuals (specificity 93 and 100%, respectively) (**Table 2**). Among 10 patients with histologically proven diagnosis of CPA, eight had a GM test result <0.5 ODI and one patient had a GM test result of 0.7 ODI, while only 1 had a GM result > 1.0 ODI.

**Table 2:** Results of ROC analysis for all patients and stratification for patients with and without COPD.

Test	All				COPD				Non-COPD			
	CPA vs. healthy		CPA vs. respiratory disorders without CPA		CPA vs. healthy		CPA vs. respiratory disorders without CPA		CPA vs. healthy		CPA vs. respiratory disorders without CPA	
	AUC	Sens and Spec (%)	AUC	Sens/Spec	AUC	Sens/Spec	AUC	Sens/Spec	AUC	Sens/Spec	AUC	Sens/Spec
GM 0.5 ODI	87.5 (78.2–96.8)	Sens: 40.7 Spec: 100	71.8 (58.1–85.5)	Sens: 40.7 Spec: 92.6	95.8% (90.2%–100%)	Sens: 45.5 Spec: 100	64.3 (40.9–87.7)	Sens: 45.5 Spec: 90	22.2% (67.8–95.8%)	Sens: 37.5 Spec: 100	80.5 (64.6–96.3)	Sens: 37.5 Spec: 100
GM 1.0 ODI	87.5 (78.2–96.8)	Sens: 29.6 Sens: 100	71.8 (58.1–85.5)	Sens: 29.6 Spec: 100	95.8% (90.2%–100%)	Sens: 45.5 Spec: 100	64.3 (40.9–87.7)	Sens: 45.5 Spec: 100	81.8% (67.8–95.8%)	Sens: 18.8 Spec: 100	80.5 (64.6–96.3)	Sens: 18.8 Spec: 100
IFN- γ	87.6 (74.2–100)	Sens: 88.0 Spec: 26.7	54.8 (30.8–78.9)	Sens: 88.0 Spec: 0	77.4 (55.5–99.4)	Sens: 88.9 Spec: 26.7	63.5 (9.0–87.9)	Sens: 88.9 Spec: 0	87.6 (74.2–100)	Sens: 93.3 Spec: 26.7	54.8 (30.6–78.9)	Sens: 93.3 Spec: 0%
IL-10	64.6 (55.3–73.9)	Sens: 63.0 Spec: 100	45.8 (32.4–59.1)	Sens: 63.0 Spec: 63.3	70.0 (54.–84.6%)	Sens: 40 Spec: 100	43.7 (24.1–63)	Sens: 40 Spec: 100	75.0 (61.4–88.6)	Sens: 21.4 Spec: 100	60.7(49.6–71.9)	Sens: 21.4 Spec: 100
IL-15	55.2 (33.5–76.8	Sens: 33.3 Spec: 80	55.8 (35.9–75.7)	Sens: 33.3 Spec: 40	71.6% (38.4%–100%)	Sens: 60 Spec: 80	55.0 (26.6–83.4)	Sens: 80 Spec: 38.9	43.3 (18–68.)	Sens: 14.3 Spec: 100	67.3 (34.9–99.8)	Sens: 85.7 Spec: 42.9
IL-1b	93.8 (86.9–100.0)	Sens: 85.1 Spec:100.0	49.6 (33.7–65.59)	Sens: 85.1 Spec: 22.2	96.1 (89.4%–100%)	Sens: 81.3 Spec: 100	55.0 (32.7–77.3)	Sens: 82.3 Spec: 28.6	92.2 (81.5–100)	Sens: 81.8 Spec: 100	62.1 (38.0–86.1)	Sens: 81.8 Spec: 10.5
IL-6	90.2 (81.9–95.5)	Sens: 70.8 Spec: 96.3	61.9 (46.0–77.7)	Sens: 70.8 Spec: 21.1	88.1 (73.2%–100%)	Sens: 73.3 Spec: 96.3	64.0 (40.7–87.4)	Sens: 73.3 Spec: 28.6	91.5 (82.0–100)	Sens. 66.7 Spec: 96.3	47.6 (22.2–73.1)	Sens: 66.7 Spec: 15.8
IL-8	95.4 (90.1–100.0)	Sens: 92.6 Spec: 70.4	63.7 (48.4–78.9)	Sens: 92.6 Spec: 0	96.5% (89.3%–100%)	Sens: 93.8 Spec: 70.4	6.8 (39.3–82.2)	Sens: 93.8 Spec: 0	94.7 (87.3–100.0)	Sens: 90.9 Spec: 70.4	62.5 (35.2–89.8)	Sens: 90.9 Spec: 0
TNF-α	73.9 (63.5–84.3)	Sens: 47.8 Spec: 100	54.1 (38.6–69.6)	Sens: 47.8 Spec: 42.3	72.2% (5.0–89.4%)	Sens: 50.0 Spec: 100	55.3 (33.7–76.8)	Sens: 50.0 Spec: 28.6	75 (61.4–88.6)	Sens: 44.4 Spec: 100	50.5 (26.4–74.6)	Sens: 44.4 Spec: 47.4


*AUC, Area under the curve; COPD, Chronic obstructive pulmonary disease; CPA, Chronic pulmonary aspergillosis; GM, Galactomannan; IFN, Interferon; IL, Interleukins; ODI, Optical density index; Sens, Sensitivity; Spec, Specificity; TNF, Tumor necrosis factor.*

The diagnostic performance of the GM test tended to be higher when COPD was present in CPA patients (sensitivity 46% vs. 38%; cut off 0.5 ODI and 46% vs. 19%; cut off 1.0 ODI respectively) (**Table 3**).

**Table 3:** *P-*values of pairwise Wilcoxon rank sum test for GM and cytokines.

	CPA vs. respiratory disorders without CPA	CPA vs. healthy individuals	Respiratory disorders without CPA vs. healthy individuals
GM	*p* = 0.013^∗^	*p* < 0.001^∗^	*p* = 0.081
IFN- γ	*p* = 0.477	*p* = 0.001^∗^	*p* < 0.001^∗^
IL-10	*p* = 0.541	*p* = 0.072	*p* = 0.057
IL-15	*p* = 1.00	*p* = 1.00	*p* = 0.14
IL-1b	*p* = 0.96	*p* < 0.001^∗^	*p* < 0.001^∗^
IL-6	*p* = 0.15	*p* < 0.001^∗^	*p* < 0.001^∗^
IL-8	*p* = 0.089	*p* < 0.001^∗^	*p* < 0.001^∗^
TNF-α	*p* = 0.609	*p* = 0.008^∗^	*p* = 0.003^∗^


*CPA, Chronic pulmonary aspergillosis; GM, Galactomannan; IFN, Interferon; TNF, Tumor necrosis factor. ^∗^*P*-value <0.05 statistically significant.*

**FIGURE 1:**
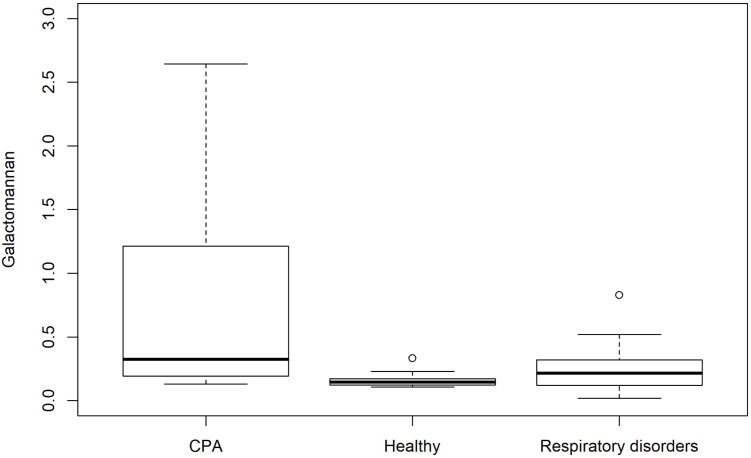
Box plot showing the distribution of GM antigen levels for patients with CPA and controls including patients with respiratory disorders without CPA and healthy individuals.

**FIGURE 2:**
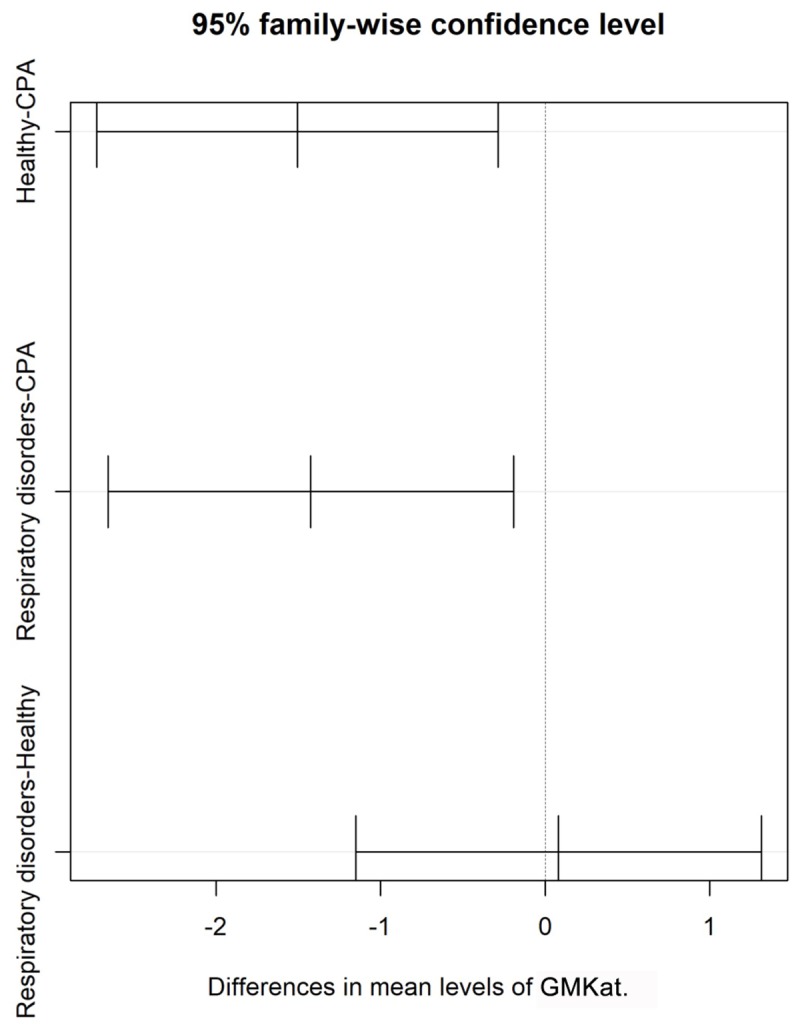
Shows differences in mean of GM antigen levels with 95% confidence interval for patients with CPA and for controls including patients with respiratory disorders without CPA and healthy individuals.

The distribution of GM test results for all three cohorts is shown in **Figure 1**. GM values were significantly higher in CPA samples compared to patients with respiratory disorders without CPA (*p* = 0.013) and healthy individuals (*p* < 0.001). No significant differences in GM values were found between healthy individuals and patients with other respiratory diseases without CPA (*p* = 0.081) (**Table 3**). Tukey test showed a significant difference in mean values for GM test between CPA patients compared to patients with respiratory disorders without CPA and to healthy individuals (**Figure 2**).

**FIGURE 3:**
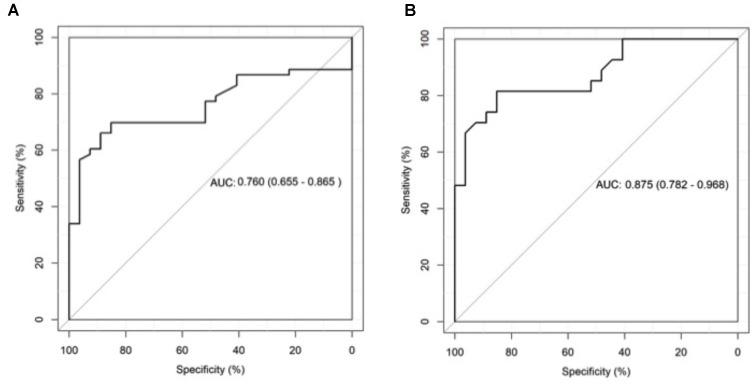
Receiver operating characteristics (ROC) curve analysis of galactomannan test results in bronchoalveolar lavage fluid (BALF) for patients with chronic pulmonary aspergillosis (CPA) and patients with respiratory disorders without CPA **(A)** as well as for patients with CPA and healthy individuals **(B)**.

ROC curve analysis showed an AUC 0.718 (95% CI 0.581–0.855) for differentiating CPA patients from patients with other respiratory diseases without CPA (**Figure 3**) and an AUC of 0.875 (95% CI 0.782–0.968) for differentiating CPA patients from healthy individuals (**Figure 3**).

### Newly Formatted Lateral-Flow Device

The *Aspergillus* LFD showed positive test results in two CPA patients (sensitivity 7%) and in one patient with respiratory disorder without CPA, but none in healthy volunteers (specificity 96 and 100%, respectively). Both CPA patients with a positive LFD had a high GM test result of 14.0 and 5.4 ODI, respectively, while the one patient without CPA had a negative GM test result of 0.38 ODI.

### Cytokines

**FIGURE 4:**
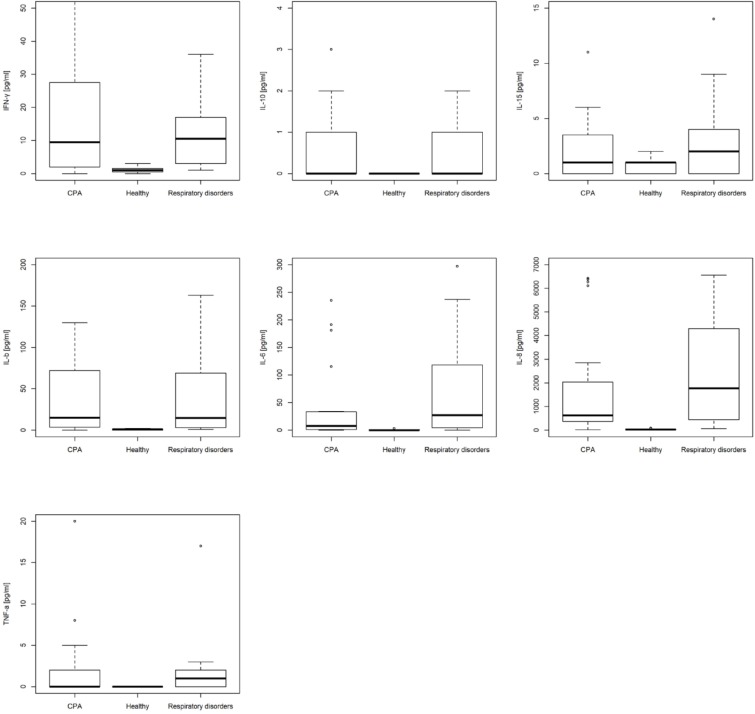
Box plots of bronchoalveolar lavage fluid (BALF) interleukin (IL)-1β, IL-6, IL-8, IL-10, IL-15, interferon-γ, and tumor necrosis factor (TNF)-α in patients with chronic pulmonary aspergillosis (CPA), patients with respiratory disorders without CPA and healthy individuals.

Median and standard deviation of CPA patients vs. controls including patients with respiratory disorders without CPA and healthy individuals are depicted **Table 1**. Box plots for IL-1β, IL-6, IL-8, IL-10, IL-15, TNF-α, and IFN-γ are depicted in **Figure 4**. AUC for BALF cytokines for differentiating CPA patients vs. controls including patients with respiratory disorders without CPA and healthy individuals are depicted in **Table 2**.

CPA patients did not differ significantly in the BALF cytokine profile compared to patients with respiratory disorders without CPA, but showed significant higher values for IFN-γ, IL-1b, IL-6, IL-8, and TNF-α compared to healthy individuals (**Table 2**). Patients with respiratory disorders without CPA had also significantly higher values for IFN-γ, IL-1b, IL-6, IL-8, and TNF-α compared to healthy individuals.

Sensitivity of IL-1b, IL-6, and IL-8 tended to be higher in patients without COPD, but at the expense of specificity. The sensitivity of TNF-α tended to be higher when COPD was present in CPA patients (**Table 2**).

## Discussion

We evaluated diagnostic performance of GM and LFD testing, which are routinely used for the diagnosis of invasive pulmonary aspergillosis (IPA), in patients with CPA compared to patients with respiratory disorders without CPA and healthy individuals. We also explored the diagnostic potential of cytokine levels in BALF in these patient cohorts.

In the present study the sensitivity of the GM test from BALF for CPA (according to ESCMID/ERS/ECMM definition) was considerably lower than previously reported with sensitivities of 30% (cut off 1.0 ODI) and 41% (cut off 0.5 ODI), respectively. Strikingly, 80% of histologically proven cases of CPA had a negative GM test result when using the 0.5 ODI cut-off (and 90% when using the 1.0 ODI cut off). Previous studies reported higher sensitivities between 77 and 92% for a cut off ≥0.5 ODI or ≥0.4 ODI (Park et al., 2011; Izumikawa et al., 2012; Kono et al., 2013; Urabe et al., 2017). We suggest that one main reason is that studies demonstrating a higher sensitivity from BALF in CPA patients may have included a considerably higher proportion of patients with SAIA (formally chronic necrotizing or semi-IPA), which is in fact an invasive form of the disease and very similar to IPA. This has been demonstrated by the study of Kono et al. (2013) who included a total of 7 patients with pulmonary aspergillosis in their analysis including five patients with SAIA (sensitivity 86%; cut-off ≥0.5 ODI). The most recent study by Urabe et al. (2017) who reported a sensitivity of 78% for BALF GM testing (cut-off ≥0.5 ODI) among 27 CPA patients did not provide information on the SAIA proportion nor on exact GM values hampering the interpretation of the data.

Another study by Izumikawa et al. (2012) reported a sensitivity of 77% (cut off 0.4 ODI) for the GM test from BALF and included 18 CPA patients. Although they did not classify CPA subtypes, inclusion criteria considered SAIA and we suggest that a considerably proportion might have had SAIA because very high GM test results between 7.3 and 14.1 ODI were observed in 7/18 patients. In contrast, in our study only 3/27 CPA patients had a GM test result >5.0 ODI. We suggest that these three patients might have in fact had SAIA supported by the fact that the two positive LFD test results from BALF in our study were seen in those patients with the highest GM test results.

The highest sensitivity of 92% for GM from BALF (cut off ≥0.5 ODI) was reported in patients with single aspergilloma (Park et al., 2011). Interestingly, 75% of all patients included in that study (*n* = 48) had hemoptysis, which is usually considered as an expression of angioinvasion by the fungus. Furthermore, 13/34 patients even had a positive GM test in serum (cut off 0.5 ODI), which was also significantly associated with hemoptysis compared to those without hemoptysis (52% vs. 9%; p = 0.02). Therefore it is highly likely that a high proportion of patients in that study might have had a more invasive stage of disease comparable to SAIA or even IPA, which was not the case for our study cohort.

Taken this together it may not be surprising that the sensitivity of GM in BALF, which was initially developed for the diagnosis of IPA, was considerably lower in our study than compared to previous study results. It has to be considered that the vast majority of patients with CPA do not have SAIA. Thus, the diagnostic performance of GM from BALF to establish the diagnosis of CPA is insufficient. However, its high specificity may help to exclude semi-invasive or invasive disease in certain cases. We suggest that patients with a high GM test are at least in a transition stage to semi-invasive or invasive disease, while patients with a non-invasive CPA subtype usually do not respond with a positive GM from BALF. Other factors that may influence the performance of GM in BALF include antifungal treatment, certain antimicrobial drugs (e.g., beta-lactam antibiotics), underlying diseases (e.g., allergic bronchopulmonary aspergillosis), or BALF sampling bias (e.g., volume and site of lavage fluid sampling). However, bronchoscopy is still indispensable and should be integrated in every diagnostic work-up, if possible. Histology (e.g., from transbronchial biopsies) is still the reference standard to differentiate between invasive and non-invasive aspergillosis and to exclude alternative diagnosis as recommended by the current guideline (Denning et al., 2016). BALF also offers the possibility to collect fungal cultures from primary site of infection and when positive, to examine antifungal *in vitro* susceptibility (Alastruey-Izquierdo et al., 2018; Godet et al., 2018).

We did not evaluate GM test in serum, because serum was only availably in a minority of CPA patients included. Furthermore, previous studies clearly demonstrated that GM testing from serum has a very low sensitivity and specificity between 23 and 67% and 64 and 85% with an ODI ≥ 0.7 in CPA patients, which is plausible considering that CPA is a localized chronic disease of the lung without angioinvasion per definition (Izumikawa et al., 2012; Shin et al., 2014; Urabe et al., 2017).

This is to our knowledge the first study investigating cytokine levels in BALF from CPA patients. Generally, CPA patients had cytokine levels in BALF that were comparable to those found in patients with respiratory disorders and suspected infection but without CPA. However, CPA patients had significantly higher cytokine levels when compared to healthy individuals. Previous genetic association studies suggested that patients with CPA might produce lower levels of IL-10 and have ongoing or higher expression of IL-1b and IL-6 leading to a pro-inflammatory response and disease progression (Sambatakou et al., 2006; Smith et al., 2014). In our analysis IL-10 levels did not differ between CPA patients compared to patients with respiratory disorders without CPA (*p* = 0.541), but tended to be higher compared to healthy individuals (*p* = 0.072), where IL-10 levels were often below the detection limit. Levels of IL-1b, IL-6, IL-8, TNF-α, and IFN-γ were significantly higher in CPA patients compared to healthy individuals (*p* < 0.001 and *p* = 0.008, respectively), but not different than in patients with respiratory disorders without CPA. This stands in contrast with previous suggestions that CPA might be associated with low levels of IFN-γ (Doffinger D, AAA2014 Abstract) and that TNF-α might be linked with aspergillosis and/or chronic cavitary pulmonary asergillosis (Sambatakou et al., 2006).

This analysis has several limitations including the small group size, however, at least in GM a power of over 0.8 was found during the subsequent power analysis. With regard to the LFD, no statement can be made about significant differences or test performance, as only three LFD tests were positive in total. Furthermore, some biomarkers have a very wide range; the mean values are partly outside the third quartile. Box plots showed that, regardless of extreme values and outliers, the values are systematically unequal (or equal) distributed across the individual groups. Furthermore, non-parametric tests were used to minimize distortions due to non-normal distribution and variance heterogeneity. Another limitation is the lack of a reference standard for the diagnosis of patients with CPA (e.g., growth in transbronchial biopsies/histopathology showing fungal elements). Although accepted by ESCMID, ERS, and ECMM the clinical definition of CPA is ambiguous and may be incorrect in a certain number of patients.

## Conclusion

Both GM and LFD showed insufficient performance for diagnosing CPA, with sensitivities of BALF GM below 50%, and sensitivity of the LFD below 10%. The high specificities may, however, result in a high positive predictive value and thereby help to identify semi-invasive or invasive disease. This has direct clinical implications, because CPA patients with SAIA have a more rapid disease progression and should be managed like patients with IPA. Any diagnostic assay to proof mycological evidence needs to be interpreted in the clinical and radiological context as recommended by current guidelines.

## Author Contributions

HS, JP, and MH contributed to the scientific literature search, study design, data collection, data analysis, and drafting the manuscript. HF, JH, BK, SO, KG, CH, and CL were involved in the material collection and data analyses. All authors were involved in revision of the paper and final approval of the version to be published.

## Conflict of Interest Statement

HS has received research grants from Gilead and honoraria for lectures from Chiesi, outside the submitted work. JP received consulting fees from Gilead, outside the submitted work. HF has received consulting fees or speakers’ honoraria from Pfizer, Roche, Novartis, Merck, Boehringer Ingelheim, PARI Pharma, and AOP, outside the submitted work. JH reports personal fees from Chiesi, Hain, Jnssen and Lucane, outside the submitted work. BK reports personal fees from Oxford Immunotec and Lucane, outside the submitted work. CH reports personal fees from AstraZeneca, Bayer, Genzyme, Insmed, outside the submitted work. GJ is the scientific director at OLM Diagnostics, but had no influence on study design, test performance, data analyses and interpretation. CL has received personal fees from Chiesi, Gilead, Janssen, Lucane, Novartis, Thermofisher and Transgene outside the submitted work. MH has received research grants from Gilead and speakers honoraria from Gilead, Basilea and Merck, outside the submitted work. The remaining authors declare that the research was conducted in the absence of any commercial or financial relationships that could be construed as a potential conflict of interest.

## Footnotes

1 https://www.mesoscale.com
